# Peripheral role of glutamate in orofacial pain

**DOI:** 10.3389/fnins.2022.929136

**Published:** 2022-11-09

**Authors:** Jinyue Liu, Shilin Jia, Fang Huang, Hongwen He, Wenguo Fan

**Affiliations:** ^1^Hospital of Stomatology, Guanghua School of Stomatology, Sun Yat-sen University, Guangzhou, China; ^2^Guangdong Provincial Key Laboratory of Stomatology, Sun Yat-sen University, Guangzhou, China

**Keywords:** glutamate, glutamate receptors, trigeminal ganglion, orofacial pain, glutamate transporters

## Abstract

Glutamate is the principal excitatory neurotransmitter in the central nervous system. In the periphery, glutamate acts as a transmitter and involves in the signaling and processing of sensory input. Glutamate acts at several types of receptors and also interacts with other transmitters/mediators under various physiological and pathophysiological conditions including chronic pain. The increasing amount of evidence suggests that glutamate may play a role through multiple mechanisms in orofacial pain processing. In this study, we reviewed the current understanding of how peripheral glutamate mediates orofacial pain, how glutamate is regulated in the periphery, and how these findings are translated into therapies for pain conditions.

## Introduction

Glutamate is one of the most abundant amino acids, which plays important roles in nutrition and metabolism in addition to its role in protein structure (Meldrum, 2000; Petroff, 2002; Brosnan and Brosnan, 2013). It is discovered as a neurotransmitter responsible for the excitatory action in the central nervous system (CNS) and involved in a wide variety of physiological and pathological processes such as learning, memory (Hugon et al., 1996), development, depression (Hillhouse and Porter, 2015), and neurodegeneration (Meldrum, 2000). In addition, it is well demonstrated that glutamate plays an important role in nociceptive processing not only at central synapses but also in the peripheral nervous system (PNS). The involvement of glutamate in nociception in the CNS has been extensively reviewed elsewhere (Fundytus, 2001; Zhuo, 2017). The increasing amount of evidence has indicated that glutamate is implicated in orofacial pain. The aim of this review was to give an overview of glutamate in the trigeminal system and analyze different lines of evidence for the role of peripheral glutamate in orofacial pain. Possible cellular mechanisms regarding the connection between glutamate and orofacial pain in the periphery are discussed.

## Nociceptive transmission of the orofacial region

The term “orofacial pain” is used to describe pain arising from the regions of the face and mouth (Ghurye and McMillan, 2017). Orofacial pain-associated disorders include but are not limited to temporomandibular muscle and joint (TMJ) disorders, jaw movement disorders, neuropathic and neurovascular pain disorders, headaches, and sleep disorders (AAOP, 2022). Pain sensation from the orofacial region is relayed to the nerve center by the trigeminal nerve system. The primary sensory neurons innervating the orofacial region are located in the trigeminal ganglia (TG, a cranial analog of the dorsal root ganglia, DRG) (Huang et al., 2013; Yudin and Rohacs, 2018), in which central processes enter the brainstem, where trigeminal second-order neurons convey the peripheral nociceptive information to the higher centers (Lazarov, 2002). Pain-sensing, primary afferent neurons of TG are classified into two main types, i.e., small diameter, slow-conducting, myelinated A-delta fibers and slow-conducting unmyelinated C fibers, which are responsible for rapid, acute pain and delayed, slow pain, respectively (Bae and Yoshida, 2020). These neurons are tightly enveloped by satellite glial cells (SGCs), and thus both form an anatomically and probably functionally distinct unit (Hanani, 2005). A variety of neurotransmitters and mediators associated with nociception are known to be present in the neurons and SGCs in the PNS (Messlinger and Russo, 2019), of which glutamate, as a transmitter, plays an important role in the etiology and pathogenesis of orofacial pain. Understanding the glutamate mechanism of orofacial pain is important for performing successful management of such painful conditions.

## Glutamatergic system in primary sensory ganglia

**FIGURE 1:**
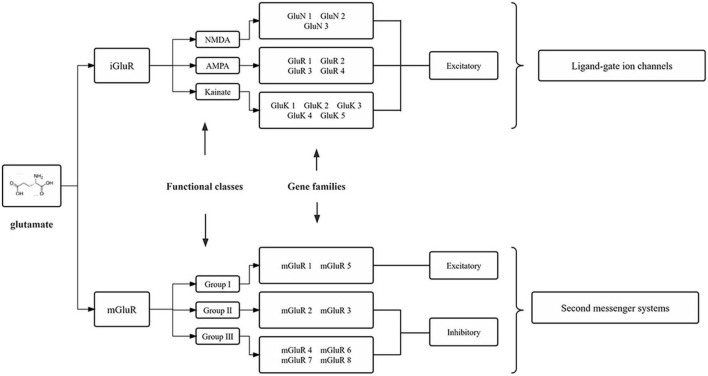
Molecular families of glutamate receptors. The two main glutamate receptors are each composed of three functional definition groups of the receptor. Numerous individual subunits, encoded by different genes, make up these receptors. iGluRs, ionotropic glutamate receptors; mGluRs, metabotropic glutamate receptors; NMDA, N-methyl-D-aspartic acid; AMPA, α-amino-3-hydroxy-5-methyl-4-isoxazole-propionic acid.

**FIGURE 2:**
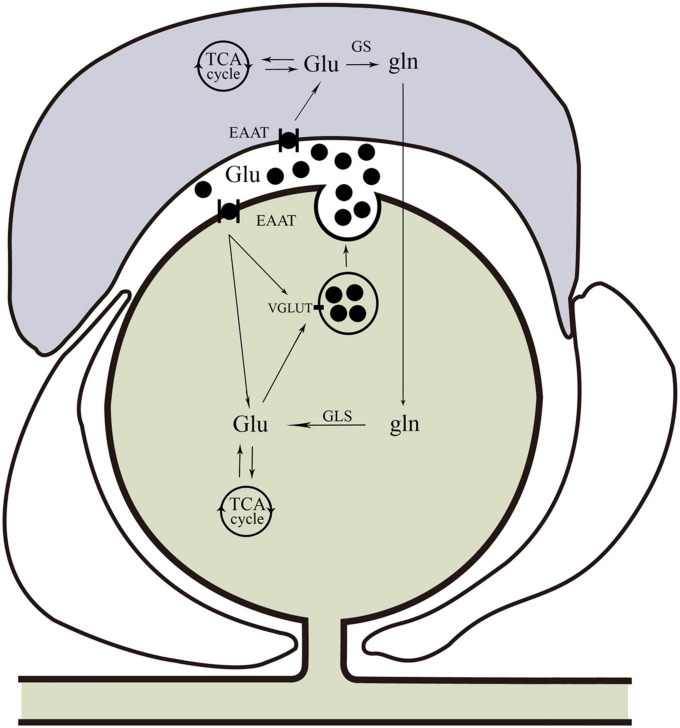
Glutamine cycle in the trigeminal nervous system. Glu can be taken up by neurons or glia. In neurons, glutamate is taken up by EAAT. Satellite glial cells take up glutamate *via* EAAT for conversion to Gln *via* GS. Glutamine can be transported back to neurons for conversion to Glu by GLS, and then Glu can be packaged into vesicles by VGLUT. In addition to the glutamine cycle, neuron and glial cells also produce glutamate *via* interactions with the neuronal TCA cycle. Glu, glutamate; EAAT, excitatory amino acid transporter; Gln, glutamine; GS, glutamine synthetase; GLS, glutaminase; VGLUT, vesicular glutamate transporters; TCA, tricarboxylic acid.

Glutamate is synthesized from the hydrolytic deamidation of glutamine as part of the glutamate-glutamine cycle by phosphate-activated glutaminase (GLS) (Miller et al., 2011). Glutamate can also be produced *via* interaction with the tricarboxylic acid (TCA) cycle (Stallard et al., 2021). In common with many other neurotransmitters, the action of glutamate is mediated *via* both ionotropic receptors and metabotropic receptors (Scheefhals and MacGillavry, 2018), which are summarized in Figure 1. There are three families of ionotropic receptors as follows: N-methyl-D-aspartate (NMDA), alpha-amino-3-hydroxy-5-methyl-4-isoxazolepropionic acid (AMPA), and kainite (KA) receptors (Bigge, 1999). There are three groups of metabotropic, G protein-coupled glutamate receptors (mGluR) as follows: Groups I, II, and III, which modify neuronal and glial excitability through G protein subunits acting on membrane ion channels and second messengers such as diacylglycerol and cAMP (Niswender and Conn, 2010). In addition to the glutamate receptor system, there are transport systems expressed both in neurons and glial cells, which not only move glutamate across the plasma membrane in both directions (e.g., glutamate uptake and exocytosis) but also refill synaptic vesicles of neurons (Olivares-Banuelos et al., 2019). The transport system includes three neuronal glutamate transporters and two glial transporters, of which excitatory amino acid transporter 3 (EAAT3), excitatory amino acid transporter 4 (EAAT4), and excitatory amino acid transporter 5 (EAAT5) are in neurons, and glutamate-aspartate transporter (EAAT1/GLAST) and glutamate transporter-1 (EAAT2/GLT-1) are in glial cells (Amiel and Mathew, 2007; Nikitin et al., 2015). For example, in SGCs, glutamate is taken up by GLAST and GLT-1 for conversion to glutamine (gln) *via* glutamine synthetase (GS) (Miller et al., 2011). Once synthesized in or taken up into neurons, glutamate is accumulated into neurotransmitter vesicles *via* vesicular glutamate transporters (VGLUTs) that are specialized proteins in the vesicular membrane (Fremeau et al., 2004; Zhang et al., 2018). There are three isoforms of VGLUT (1, 2, and 3), of which VGLUT1 and VGLUT2 are in neurons and peripheral axons (Gegelashvili and Bjerrum, 2014). Glutamate-sensing and -transporting systems seem to play important roles in molecular mechanisms underlying different pathological processes including chronic pain (refer to Figure 2).

## Involvement of glutamate in orofacial pain

The early morphological studies showed that glutamate was present in neurons of TG in the rat (Wanaka et al., 1987; Csati et al., 2015) and cat (Lee and Ro, 2007a). Approximately 30–80% of total neurons in TG are glutamatergic (Kai-Kai and Howe, 1991; Li et al., 2003). Glutamate receptors (ionotropic and metabotropic) are found on the cell membranes of the primary sensory neuron of TG. Ionotropic glutamate receptors (iGluRs) including NMDA (NR1, NR2A, NR2B), AMPA (GluR1 and GluR2), and KA receptors (such as GluK5) occur in TG neurons (Sahara et al., 1997; Lee and Ro, 2007a; Chun et al., 2008; Csati et al., 2015). In addition, NMDA subunit NR2A, KA receptor subunit GluK2, and AMPA subunit GluR4 were immune-labeled in SGCs (Tachibana et al., 1994; Kung et al., 2013). Group I metabotropic glutamate receptors (mGluRs) (mGluR1α and mGluR5), group II (mGluR2 and mGluR3), and group III (mGluR8) mGluRs occur in TG neurons, while SGCs only express mGluR1α and mGluR8 (Lee and Ro, 2007a,b; Kim et al., 2009; Boye Larsen et al., 2014). All three types of ionotropic glutamate receptors and mGluR1α and mGluR8 have been observed in SGCs (Boye Larsen et al., 2014; Fernandez-Montoya et al., 2017). Moreover, the machinery for the production, release, and recycling of glutamate is present in the trigeminal nerve including the glutaminase (GLS) (Hoffman et al., 2016), vesicular glutamate transporters (VGLUT1, 2, and 3) (Kaneko and Fujiyama, 2002; Kim et al., 2015, 2018; Wang et al., 2016; Zhang et al., 2018), the GLAST, and GLT1 (Malik and Willnow, 2019), as well as the recycling enzyme GS (Miller et al., 2002; Weick et al., 2003). The anatomical distribution of the glutamatergic system in TG indicates that glutamate can be an important factor in orofacial pain.

Substantial evidence exists in support of the peripheral glutamatergic system playing important roles in orofacial pain. Myofascial temporomandibular disorders (TMDs) are the most common cause of chronic pain in the orofacial region. A clinical study showed that there were differences in the masseter muscle levels of glutamate during acute nociception in patients with myofascial TMD compared with healthy subjects (Bajramaj et al., 2019). Injecting glutamate into the human masseter muscle or temporomandibular joint causes acute pain and/or mechanical allodynia (Svensson et al., 2005; Castrillon et al., 2012; Suzuki et al., 2017; Exposto et al., 2018; Udagawa et al., 2018; Shimada et al., 2020; Alhilou et al., 2021a), which is confirmed in animals (Cairns et al., 2002; Lam et al., 2009; Jie et al., 2018). When NMDA is injected into the rat masseter muscle, an increase occurs in muscle afferent discharge in a dose-related manner (Dong et al., 2007). Another study showed that increased peripheral glutamate receptor (NMDA-receptor) expression partly contributed to masseter muscle pain sensitivity induced by intramuscular injection of nerve growth factor (NGF) in healthy humans (Alhilou et al., 2021a,b). Other studies indicate that there are increased expression levels of glutamate receptors in the TG in orofacial pain induced by inferior alveolar (or infraorbital) nerve injury (Li et al., 2020, 2021; Kurisu et al., 2022) and NGF (Wong et al., 2014). Application of glutamate receptor antagonists reduced nociceptive trigeminal responses in different orofacial pain models (Lam et al., 2005; Lee and Ro, 2007b; Li et al., 2013; Csati et al., 2015). The data suggest that peripheral glutamate may activate peripheral nociceptive afferents *via* its receptor, resulting in orofacial pain. Endogenous sources of glutamate in the periphery include plasma, macrophages, epithelial and dendritic cells in the epidermis and dermis, odontoblasts (Cho et al., 2016; Nishiyama et al., 2016), and Merkel cells (MCs) (Higashikawa et al., 2019). Glutamate is released from the mechanically stimulated odontoblast into the extracellular space *via* glutamate-permeable anion channels, and higher levels of glutamate are linked to increased sensations of pain (Cho et al., 2016; Nishiyama et al., 2016). Glutamate increases in the TG following a chronic constriction injury of the inferior orbital nerve, which can be released within the TG and can contribute to nociception (Kung et al., 2013). Experimental traumatic occlusion causes a long-lasting nociceptive response, in which the release of glutamate increases, and AMPA and NMDA receptors are upregulated in the TG (Abdalla et al., 2022). Since EAATs are expressed by SGCs in the TG (Miller et al., 2011; Laursen et al., 2014), it is likely that SGCs participate in regulating intra-ganglionic glutamate levels. Increased glutamate within the TG evokes afferent discharge and significantly reduces muscle afferent mechanical threshold. The glutamate-evoked discharge is attenuated by an NMDA receptor antagonist (Laursen et al., 2014). Recent studies showed that glutamate was released from SGCs and could potentially play a role in trigeminal sensory transmission (Wagner et al., 2014; da Silva et al., 2015). These data indicate that glutamate contributes to the initiation and/or maintenance of orofacial pain.

## The interaction of glutamate and other neurotransmitters/neuromodulators in orofacial pain

Inflammatory pain is initiated by tissue damage/inflammation and neuropathic pain by nervous system lesions (Latremoliere and Woolf, 2009). The mechanisms underlying chronic inflammatory and neuropathic pain pathology involve peripheral and central sensitization characterized by increased excitability of primary sensory and central neurons, respectively (Woolf and Salter, 2000). The central mechanisms of glutamate involved in pain have been well reviewed elsewhere (Fundytus, 2001; Zhuo, 2017). The mechanisms of peripheral glutamate in acute and chronic inflammatory and neuropathic pain were reviewed as follows.

Transient receptor potential (TRP) ion channels mediate various types of sensory reception such as pain, temperature, different kinds of tastes, pressure, and vision (Chung et al., 2011). Several members of the TRP family, such as transient receptor potential vanilloid 1 (TRPV1) and transient receptor potential ankyrin 1 (TRPA1), play essential roles in pain sensation (Chung et al., 2011). Approximately 30% of TG neurons innervating the lateral facial skin expressed both TRPV1 and TRPA1, and approximately 64% of TRPA1-positive neurons also expressed TRPV1 (Zurborg et al., 2007; Akopian, 2011; Honda et al., 2014). TRPA1 may serve as a downstream target of pro-nociceptive ion channels and mediate acute muscle mechanical hypersensitivity induced by glutamate (Asgar et al., 2015). Capsaicin, a TRPV1 agonist, produces acute nociceptive behaviors when injected into the mouse upper lip (de Oliveira et al., 2020). Nifedipine can suppress nociceptive behavior through the NMDA receptor system (de Oliveira et al., 2020). In addition, sensitization of TRPA1 and/or TRPV1 through mGluR5 signaling *via* PKCε is involved in facial thermal and mechanical hypersensitivity (Cesare et al., 1999; Lee and Ro, 2007b; Chung et al., 2015; Honda et al., 2017). NMDA receptor subunits and TRPV1 are colocalized in masseter afferents, and NMDA treatment sensitizes capsaicin responses in dissociated TG neurons (Lee et al., 2012; Chung and Ro, 2020). Masseter injection of NMDA increases serine phosphorylation of TRPV1 (Lee et al., 2012; Chung and Ro, 2020). The data suggest that crosstalk between glutamate receptors and TRP channels contributes to the development and maintenance of orofacial pain. Clinically, topical application of capsaicin is used for reducing orofacial pain (Epstein and Marcoe, 1994; Campbell et al., 2017), the mechanisms of which might be attributed to central inhibitory pathways triggered by the painful stimuli of capsaicin (Campbell et al., 2017). Since glutamate signaling pathways functionally interact with TRP channels, whether such mechanisms are operative in orofacial pain needs to be confirmed further.

Adenosine is an endogenous purinergic nucleoside present in many cells (Ciruela et al., 2011). Inside the cell, adenosine is formed from ATP, cAMP, or S-adenosyl-L-homocystein, while outside the cell, it arises from equilibrative nucleoside transporter-mediated release or metabolism from ATP or cAMP (Sawynok and Liu, 2003). In the periphery, adenosine is released from both neuronal and non-neuronal sources (Guieu et al., 1996; Liu et al., 2001). Adenosine can activate signaling pathways through their receptors such as A1R, A2AR, A2BR, and A3R, which can act as a neuromodulator, and is involved in pain transmission and sensitization (Chen et al., 2013). Other studies indicate that the administration of glutamate evokes peripheral adenosine release and that NMDA and AMPA receptors are involved in such release. The released adenosine may provide a negative feedback control on nociception (Zahn and van Aken, 2000; Liu et al., 2002).

Calcitonin gene-related peptide (CGRP) is a peptide neurotransmitter and is expressed in TG neurons (Edvinsson et al., 2018). Moreover, CGRP receptors are composed of the calcitonin receptor-like receptor (CLR), the receptor activity-modifying protein 1 (RAMP1), and the receptor component protein (RCP) (Messlinger et al., 2020). Overall, the expression pattern of CGRP and CGRP receptors in the TG is consistent with a model of CGRP signaling in which C-type sensory neurons release CGRP from the soma, and CGRP acts on receptors on Aδ-type sensory neurons and SGCs to modulate pain sensitivity and transmission within the ganglion (Ambalavanar and Dessem, 2009; Goto et al., 2017; Edvinsson et al., 2018). CGRP is expressed in a considerable number of VGLUT pulpal axons in humans and rats (Cho et al., 2021). The activation of AMPA and KA receptors in dental pulp may contribute to the peripheral release of CGRP, which mediates a neurogenic component of inflammation and, thus, may involve in the development of inflammatory pain (Jackson and Hargreaves, 1999), but Andreou et al.’s study showed that the activation of the peripheral iGluR5 KA receptors was able to inhibit neurogenic dural vasodilatation probably by inhibition of prejunctional release of CGRP from trigeminal afferents (Andreou et al., 2009). The different regulation of CGRP release by KA receptors remains to be established.

Nitric oxide (NO) is a free radical gas that has been shown to be produced by nitric oxide synthase (NOS) and recognized to act as a neurotransmitter or neuromodulator in the nervous system (Saha and Pahan, 2006), which may play an important role through multiple mechanisms in pain processing (Fan et al., 2012). Activation of NMDA receptors can induce NO production and release from the SGCs (Li et al., 2008; Laursen et al., 2013). Such release of NO may provide a negative regulation to NMDA receptor signaling (Fan et al., 2012). In addition, blockade of peripheral NMDA receptors significantly reduces mustard oil- and hypertonic saline-induced nocifensive behaviors, i.e., edema formation in the masseter (Ro, 2003), which suggests that glutamate may contribute to tissue edema and enhance inflammation and pain.

Repeated acute injury, neuroinflammation, and modification of pain processing systems have been linked to the development of chronic pain states (Woolf and Salter, 2000). Alterations of glutamatergic receptors and transporters in TG following damage of peripheral tissues and sensory nerves act partly on the periphery and partly on the CNS and contribute to peripheral and central sensitization. The development of acute to chronic pain is a progressive and complex process (Pak et al., 2018). Targeting the peripheral glutamatergic system may result in novel treatment options for the prevention and/or treatment of orofacial pain.

## Clinical applications

The glutamatergic system plays a key role in the pathogenesis of orofacial pain. Glutamate was injected into the masseter muscle in healthy participants and elicited pain and muscle-referred sensations (Suzuki et al., 2017; Exposto et al., 2018; Udagawa et al., 2018; Shimada et al., 2020). Masseteric injection of NGF and glutamate increased expression of peripheral NMDA receptors and could be associated with masseter muscle pain sensitivity in healthy humans (Alhilou et al., 2021a,b). The levels of glutamate in the masseter muscle during acute nociception in healthy subjects and in patients with TMD are different (Bajramaj et al., 2019). Inhibition of glutamate release, or of glutamate receptors, in the central or periphery attenuates orofacial pain in animal models. The idea that glutamate does act as a therapeutic target for pain has been tested in some clinical trials. However, the results have been inconsistent. Antagonists at NMDA and AMPA/KA receptors, such as intra-articular ketamine injections in patients with TMD, have been used clinically with some limited success (Fundytus, 2001; Ayesh et al., 2008). Metabotropic GluRs play a vital role in pain (Lee and Ro, 2007b; Chung et al., 2015; Seven et al., 2021) and may therefore be a potent new target for future drug development. In addition, the development of more specific GluR inhibitors such as antibodies or antisense oligonucleotides is highly demanded. Local delivery of GluR inhibitors or siRNA strategy may also be considered.

## Conclusion

**FIGURE 3:**
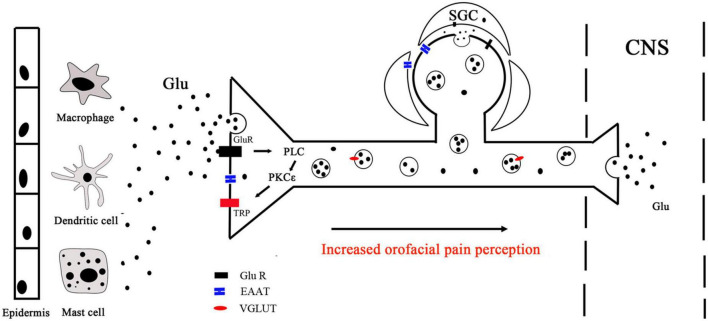
Peripheral glutamate mechanisms. Peripheral glutamate plays an important role in the transmission of nociception under normal and pathological conditions. Macrophages, mast cells, and dendritic cells in the epidermis and dermis are important endogenous sources of glutamate, which can be transported by EAAT. Glutamate binds to receptors and activates PLC, leading to intracellular Ca^2+^ release and PKCε activation, which ultimately activates TRP ion channels to mediate peripheral sensation. TG neurons store glutamate in vesicles (black circles) for release in the peripheral and central nervous. With the presence of noxious stimulation, nerve endings release glutamate stored in vesicles into peripheral tissues. Glutamate released from the same or a nearby terminal can interact with Glu R to activate or sensitize the terminals. CNS, central nervous system; PLC, phospholipase C; PKCε, protein kinase C epsilon; Glu R, glutamate receptor; TRP, transient receptor potential; SGC, satellite glial cell.

The neuronal and non-neuronal release of glutamate and their implications for orofacial pain signaling are summarized in Figures 2, 3. The excitatory amino acid glutamate and the receptors on which it acts play an important role in orofacial nociceptive processing. The regulation of the glutamatergic system is crucial not only in the CNS but also in the PNS to prevent disturbances in sensory transmission. The importance of glutamate as a neurotransmitter in the CNS is well documented, but less is known about the regulation of glutamate in the PNS, compared with the CNS, especially in the trigeminal nervous system. The progress in understanding glutamate in the CNS would be helpful for exploring the glutamate mechanisms in the PNS. Similarly, the data about the role of glutamate obtained in trunk and limb pain could also be operative in orofacial pain, which would be worthy to be confirmed, although the pathophysiology of the trigeminal nerve is in many ways different from that found in spinal nerves (Fried et al., 2001; Latremoliere et al., 2008). Regulating the glutamatergic system in the trigeminal nerve may provide a viable new target for treating orofacial pain. A comprehensive understanding of the underlying mechanisms for the observed effects of the glutamate system in the periphery will be necessary before their use can be evaluated in clinical applications for the prevention and/or treatment of orofacial pain.

## Author contributions

JL and WF contributed to conception and design of the study. JL wrote the first draft of the manuscript. WF, SJ, FH, and HH wrote sections of the manuscript. All authors contributed to manuscript revision, read, and approved the submitted version.
